# Sexual Health and Women Living With Spinal Cord Injury: The Unheard Voice

**DOI:** 10.3389/fresc.2022.853647

**Published:** 2022-05-06

**Authors:** Jennifer Ann Piatt, Ivanka Simic Stanojevic, Cedomir Stanojevic, Melissa L. Zahl, Mary Ann Richmond, Debra Herbenick

**Affiliations:** ^1^Department of Health and Wellness Design, School of Public Health, Indiana University, Bloomington, IN, United States; ^2^Department of Occupational and Recreational Therapies, College of Health, University of Utah, Salt Lake City, UT, United States; ^3^Veterans Affairs Northeast Ohio Health Care, Cleveland, OH, United States; ^4^Department of Applied Health Sciences, School of Public Health, Indiana University, Bloomington, IN, United States

**Keywords:** women's sexual health, spinal cord injury, psychosocial dimension, sexual function, disability

## Abstract

Women's sexual health within the context of sexual function and psychosocial dimensions while living with a spinal cord injury (SCI) has rarely been discussed separately from men living with a SCI or from a collective with other chronic conditions. To date, over 64,000 women in the U.S. are currently living with SCI, with total numbers increasing each year, as well as the demographics shifting to include more diversity in race and incidences occurring later in life. On average, SCI tends to be acquired during the childbearing years (~30–50 years old), as well as when women experience other health concerns associated with aging, including perimenopause and menopause. Additionally, women's sexual health is often conceptualized from the position of the absence of disease and dysfunction. However, consistent with definitions furthered by the World Health Organization (WHO) and World Association of Sexual Health (WAS), we believe women's sexual health is multifaceted, moving beyond a focus on reproduction to also encompass sexual function and the psychosocial dimensions of sexual health both living with and without disabling conditions and diseases. Within this lens, we present prior research that has been conducted, conclusions from these studies, implications for practice, and recommendations for future research. Thus, the paper will expand the understanding of both sexual function and psychosocial dimensions for women living with SCI.

## Introduction

According to the WHO (1), health impacts quality of life beyond the absence of illness or disease, and rather encompasses an interaction between biological and psychological factors, and the social environments in which one lives. Sexual health also has multiple dimensions (e.g., biological, psychological, cultural, relational) and is important to be included in rehabilitation efforts. Attending to the sexual health and sexual function of women living with SCI supports whole-person care for these women, which will improve clinical outcomes and decrease health care costs. Collectively, our research team conducted an extensive literature search on what research has been conducted within the lens of sexual health and women living with a SCI. Most of the literature and research these authors found focused on men or includes both men and women together in the discussion. Three of the research team members utilized keywords (women's sexual health, spinal cord injury, psychosocial dimension, sexual function, disability) through the database search engines of Indiana University to identify research focused on women's sexual health and living with a SCI. The authors met as a research team to eliminate those articles that did not pertain to the focus of this manuscript: women's sexual health and living with a SCI. Following is the most relevant research our team identified that addresses this topic.

Sexual function and associated psychosocial dimensions for women living with SCI have rarely been examined within the research, nor discussed in clinical practice. To date, over 64,000 women in the U.S. are currently living with SCI, with numbers rising every year. On average, SCI diagnoses tend to be acquired in the middle adult years (~30–50 years old), when women are at the prime age of their childbearing years, are anticipating major life changes (i.e., pregnancy, hysterectomy, perimenopause, menopause), or are experiencing other health concerns associated with typical aging that may affect their sexual behaviors. Prior research has investigated women's sexuality and living with SCI from the viewpoint of reproduction (2, 3), orgasm (4), and the physical act of sex (5–7). Additionally, women's sexual health is often conceptualized from the position of the absence of disease and dysfunction (8). However, consistent with definitions furthered by the World Health Organization (4) and World Association of Sexual Health (9), we believe that women's sexual health is multifaceted and moves beyond reproduction and the mechanics of sex to encompass both sexual function and psychosocial dimension.

Typically, the traditional approaches to sexual health and SCI have been rooted in the conventional medical model, including both men and women or a collective of chronic conditions, which does not observe sexual health within the lived psychosocial experiences that encompasses women's emotional needs, interpersonal relationships, body image, and cultural beliefs and values as well as their experience with their disability. Some studies tackle this multifaceted phenomenon and have identified women's sexual health as a combination of understanding of the human body, experiencing satisfaction (alone or with consenting others) while maintaining autonomy, minimizing exposure to infection, and preserving safety (10, 11). Others characterize women's sexual health including sexual satisfaction, sexual function, cognitive flexibility, sexual health/risk, attitudes and outlook, positive emotions, sexual self-esteem, and mutual and synchronous experiences (12, 13). Prior research has examined women with SCI separately (14–16), yet anecdotally we know that often health care practices will include women and men together within the sexual health programs, support groups, and educational forums. We recognize the need for the translational research aimed to understand how women, regardless of sexual orientation, with SCI identify sexual health differently than men. Most importantly research needs to consider both sexual function and psychosocial dimensions of women separate from men to better drive clinical practice.

## Women With SCI

Spinal cord injury (SCI) refers to some type of damage to the spinal cord that can result from trauma or illness. Function depends on level and type of injury as well as pre-existing conditions (17). The demographics of the SCI population has shifted slightly within the past few years. The percentage of women who acquire a SCI has recently increased from 20 to 22%. In addition, the average age of sustaining a SCI has increased from 29 to 43 years old and 24% of SCIs occur to individuals who identify as Black or Brown (non-Hispanic), which is higher than the general population of 13% (18). These shifting demographics make it more important than ever to better understand the psychosocial dimensions of sexual health, especially as related to culture and race as well as those who identify as women. Current and past relationships, divorce, dating, raising children, experiencing perimenopause and menopause, as well as other health conditions associated with typical aging are being navigated by women with SCI while learning to be a sexual being within a body that looks, moves, and feels different than prior to acquiring the SCI (18).

Coupled with all these factors, in general, the individual living with the SCI is also learning how to manage secondary health conditions (SHC) and its impact on intimate relationships. Although SHC associated with a SCI are unavoidable, the importance of managing them and understanding their impact on sexual health is crucial to a high quality of life. Prior research has demonstrated that SHC associated with the SCI do directly impact social participation (engagement in activities social in nature both in and outside of the home) (19), daily life, and intimate relationships (20).

## Women's Sexual Health and Sci

It is well documented, in prior research, men's sexual health is an important component of quality of life while living with a SCI (21), and this is no different for the women living with SCI (14, 15). Although women have unique needs related to function and psychosocial dimensions, most of the women's perspective in sexual health and SCI research is limited or clumped in with men's perspectives on their sexual health. Prior studies that have separated out men and women have shown that it was rare for women living with a SCI to have a pleasant experience with sexual desire, body image, emotional closeness, or the ability to communicate sexual needs (22). Yet, sex is identified as one of the most important topics for women post-acquisition of a SCI; often before asking the question “can I walk again”, individuals ask “can I have sex again” (14, 15). In previous research, women living with a SCI expressed sexual needs change post diagnosis and the psychosocial connection often takes precedence over physical sexual needs, including reproduction, orgasm, and the physical act of sex (16, 23). Our previous study showed that women with SCI desire the emotional connection with their partner over physical intercourse (24). It is fundamental that women living with SCI have relationships (sexual in nature) that are built on realistic expectations and openness to communication (25), rather than false assumptions that arise from popular media and lacking information based on evidence.

Additionally, prior research identified sexuality and intimacy as one of the important topics for individuals living with a SCI (14, 15) and their partners. Sexual health has been identified as the main priority for the individual living with paraplegia and the second most important (first being function of their arms) for individuals with tetraplegia (15). Early research attended to sexual response and orgasm (4), and reproductive health (3) among women living with SCI. More current research has demonstrated the importance of intimacy-based motivations and of women's mood pre- and post-sex (26). Yet to be explored are the sexual health needs of partners and/or significant others of the women living with SCI. Although there has been research on sexual health needs of the individual post acquisition of the SCI, little to no research is being conducted on the function and psychosocial dimensions of the partners' sexual health needs.

To further complicate this lack of addressing sexual health in women with SCI and their partners, the decrease in rehabilitation hospital stays from 98 days in 1974 to 30 days in the United States currently has made the information provided untimely (too early within treatment) with no follow-up during outpatient rehabilitation (18). In many cases, the topic of sexual health is presented when women may not be ready to discuss sexual health and are still adjusting to their newly acquired injury or diagnosis. According to our preliminary work, sexual health is typically not addressed again during follow-up care unless the patient seeks out specific information from a health care provider or engages in independent internet-based research. Of special note, our prior research indicated that women also desire having the information on sexual health be endorsed by a rehabilitation hospital or an institution of education, so they are assured the information is accurate and evidence based (24, 27). Taken together, this means that we need to find ways to connect with patients on sexual health topics at developmentally appropriate times in their rehabilitation. Recent research also supports this need for sexual health rehabilitation framework to drive health care practices (15).

Sexual function and psychosocial dimensions are important, regardless of whether there is a SCI present or not. The conversation becomes even more important when adjusting to a body that looks, feels, and moves differently. Understanding sexual function and the psychosocial dimensions of sexual health among women living with SCI is critical toward developing clinical and educational interventions to support these women's sexual health, relationships, and overall quality of life (28). We know positive sexual health is linked to greater life satisfaction, greater relationship satisfaction, and improved physical and mental health (29–34). In another study with the general female population when psychosocial dimensions were accounted for, mild depressive symptoms increased the risk of female sexual dysfunction (35). Since individuals living with SCI are more prone to experience depression, anxiety, and higher suicidal rates than the general population (36–38), we believe improving sexual health resources will have a positive impact on psychosocial health outcomes. Indeed, examining how these women define sexual function (e.g., desire, arousal, lubrication, orgasm, satisfaction, pain) and associated psychosocial dimensions (e.g., adjustment to disability, self-esteem, emotional, interpersonal relationships, body image, cultural beliefs and values) is necessary to understand sexual health for women with SCI.

To drive health care practice and future research there needs to be a better understanding of sexual function and associated psychosocial dimensions of women living with SCI to include women of all sexual orientations and racial identities. Since 2012, this research team has been examining sexual health and living with a disability (20, 21, 24, 27, 39–41). We also believe it is important to understand the psychosocial dimensions of sexual health unique to women that include, but are not limited to, body image (42), cultural beliefs (religion, race, nationality) (43–45), and values (defining what is important sexually) (46). Although prior research has touched on this, the evidence to drive practice is narrow and limited, preventing health care to fully address the sexual needs of women with SCI, thus addressing the entire health of women with SCI. Better understanding women's sexual health from the context of living with a SCI will help better inform health care providers within rehabilitation who are currently addressing the sexual health needs (i.e., occupational therapists, recreational therapists, nurses, social workers) so that clinical practice is up to date and can better address clinical outcomes.

## Discussion

Current research and rehabilitation practice concerning the sexual health of women living with SCI is limited and does not address the multifaceted context of sexual health. Considering the relevant impact of psychosocial dimensions on quality of life and rehabilitation practices, we believe a better understanding of women's sexual health psychosocial dimensions is necessary to improve health care practices for women with SCI. With SCI being associated with a unique subset of SHC, it is unclear if sexual function post acquisition of injury or diagnosis impacts clinical outcomes in the same manner when accounting for psychosocial dimensions in relationship to SCI. Prior studies involving women have identified rare positive experiences of sexual desire, body image, emotional closeness, and the ability to communicate sexual needs while living with an SCI (22). However, it is unclear how women explain the lived experience of living with SCI and what psychosocial dimensions drive their clinical outcomes associated with sexual function (47). Additionally, it is unclear how this impacts their intimate partner's sexual health.

Nevertheless, only minimal sexual health education occurs during medical school or during the allied health professions trainings, lending to the importance of better understanding these concepts from the women's voice to improve rehabilitation practices that lead to a greater quality of life (48). Additionally, since negative psychosocial experiences and emotions can accelerate a variety of health threats (e.g., depression, anxiety, isolation), addressing these concerns specific to women with SCI will help advance the delivery of sexual health interventions and education throughout the lifespan of women living with SCI. Indeed, addressing and supporting the continuation of healthy sexual behaviors and practices of women with SCI, healthcare providers could in fact reduce onset of SHC, bringing a more stable clinical picture of the debilitating condition. As the result, this may decrease the number of clinical visits and thus reduce healthcare costs of these individuals.

## Author Contributions

JP was the lead author conceptualizing the idea for the paper, providing content, leading the team in the writing and aproved the final version to be published, and is accountable for all aspects of the work. IS, CS, and MZ drafted the manuscript and critically revised the manuscript for important intellectual content, approved the final version to be published, and agreed to be accountable for all aspects of the work. MR and DH helped with developing the concept of the paper, contributed to the original draft, provided expert advice, and edits through the process. All authors contributed to the article and approved the submitted version.

## Funding

This work was supported by the internal funds through Indiana University, Department of Health and Wellness Design.

## Conflict of Interest

The authors declare that the research was conducted in the absence of any commercial or financial relationships that could be construed as a potential conflict of interest.

## Publisher's Note

All claims expressed in this article are solely those of the authors and do not necessarily represent those of their affiliated organizations, or those of the publisher, the editors and the reviewers. Any product that may be evaluated in this article, or claim that may be made by its manufacturer, is not guaranteed or endorsed by the publisher.
